# Challenges facing international students at Iranian universities: a cross-sectional survey

**DOI:** 10.1186/s12909-024-05167-x

**Published:** 2024-03-01

**Authors:** Maryam Tajvar, Elahe Ahmadizadeh, Haniye Sadat Sajadi, Iyad Ibrahim Shaqura

**Affiliations:** 1 Department of Health Management, Policy and Economics, School of Public Health, Tehran University of Medical Sciences, Tehran, Iran; 2 Knowledge Utilization Research Center, University Research and Development Center, Tehran University of Medical Sciences, Tehran, Iran; 3 Quality Department, Palestinian Ministry of Health, Gaza, Palestine

**Keywords:** International students, Challenges, Iranian universities, Support services, Academic success

## Abstract

**Background:**

This study examines the challenges faced by international students at Tehran University of Medical Sciences (TUMS) and provides insights into their experiences and needs.

**Methodology:**

A self-developed structured questionnaire was administered to international students who completed their first year at TUMS. Data were collected from 165 participants (76% response rate) and analyzed using SPSS 22.0.

**Results:**

The study reveals that international students encounter various challenges, including difficulties accessing information, language barriers, ineffective communication, administrative inefficiencies, cultural issues, financial constraints, and limited scholarship opportunities. Analysis of students’ characteristics indicates that the length of study at TUMS is significantly associated with the challenges experienced. The findings highlight the importance of enhancing support services and resources for international students, such as language classes, academic counseling, scholarships, and cultural exchange programs.

**Conclusion:**

Addressing these challenges can foster a more supportive environment, contributing to the academic success and well-being of international students at TUMS and similar universities.

## Introduction

International students face unique challenges when studying in universities abroad, and these challenges can vary depending on a number of factors, such as the country they are studying in, the language of instruction, the culture and social norms of the host country, and their financial situation [1]. Universities across the world have become increasingly diverse, with students from different cultural and linguistic backgrounds coming together to study in an international academic environment [2]. However, with this diversity comes the need for universities to provide adequate support to help international students overcome the challenges they may face [3].

The past decade has seen a significant growth in enrolment by international students worldwide. According to UNESCO’s Institute for Statistics, the number of students in Europe and Northern America increased by 24% between 2000 and 2020. In 2020, the largest share (about a third) of higher education students worldwide were enrolled in Eastern and South Eastern Asia (nearly 77 million students) [4]. This trend reflects the increasing globalization of higher education, as students seek to broaden their horizons and gain valuable academic and cultural experiences. However, studying in a foreign country can also be a daunting experience, particularly for international students who may face language barriers, cultural differences, academic issues and financial constraints [5]. These challenges can have a significant impact on the academic performance, mental health, and overall well-being of international students [5]. It is therefore essential for universities to recognize the unique needs and experiences of international students and provide the necessary support to help them succeed academically and personally [2].

Iran is one of the countries with rising trend of internationalization of its universities. In recent years, Iran has been attracting an increasing number of international students. According to the Iranian Ministry of Science, Research, and Technology, the number of international students studying in Iran reached about 57,000 in the academic year 2020–2021 [6]. This indicates a growing appeal of Iranian universities among international students. In addition, Iranian universities have been actively establishing partnerships and collaborations with universities and institutions around the world, and also have been increasing the number of English-taught programs to attract international students [7]. Therefore, it is imperative to take consideration on the possible challenges that current international students might experience during their studies at the Iranian universities [8].

As such, this study focuses on the challenges facing international students studying in an Iranian university, Tehran University of Medical Sciences (TUMS). TUMS, the first modern center of medical training in Iran, has been founded in 1851, and it is the oldest and most well-known medical center in Iran, nationally as well as internationally [9]. TUMS has many teaching hospitals and, as one of the country’s top research universities, accepts applications in medical sciences from the most qualified students. TUMS is a large university. One of the many advantages of its size is that it can offer a wide range of courses. Admission to TUMS is granted without regard to race, color, religion, gender nor nationality [9]. At the time of data collection for this study, TUMS had 862 enrolled students (61% men) from 40 countries, mainly from Iraq (24%), India (23%) and Pakistan (19%), half of them were Medical Doctor (MD) students, and the next popularity was Dentistry (17%), of which 78% enjoyed partial or full scholarship [9].

Literature review on this topic showed that there is very sporadic studies conducted in Iran and nothing in TUMS. Thus, there was little information on the type and frequency of challenges experienced by international students in Iran and TUMS. However, as the faculty member of TUMS sometimes we were witnessing complaints by international students about their various challenges and barriers. On the other hand, extending the internationalization of TUMS has always been one of the high priorities of this university. These altogether encouraged us to examine these challenges in TUMS, for gaining a better understanding of the distinct experiences and needs of international students, and identify strategies that universities including TUMS can use to better support them.

## Methods

### Study design and setting

This is a descriptive- analytical cross-sectional study which was conducted among the international students at TUMS which opens its doors to a wide array of majors for undergraduate and graduate students. It should be noted that no intervention was directed to improve the situation of students’ life during the study span.

### Sampling and sample size

All international students, who have been enrolling at TUMS, undergraduate or graduate who had finished their first year, and those who were willing to participate, living in dormitories or personal accommodations, have been included in the current study. Accordingly, the targeted number of students at the time of study was about 500. Of them, 20 have been recruited for the pilot study to pretest the questionnaire used for data collection, and 218 were drawn as the sample size of the study. Domestic students, international students who had not finished their first years at TUMS, those who were studying at any university other than TUMS, or alumni were excluded.

### Data collection

Data collection was performed using a structured questionnaire. For developing this questionnaire, firstly we reviewed the literature extracted from similar studies, for providing a list of possible student challenges. Then we hold a focus group discussion (FDG) with 15 students with maximum variation of those who were eligible to participate in this study. We initially asked them to have a look at the result from literature review to help them to remind all the possible challenges that they have personally experienced since formal application to the TUMS till now. During the FDG and profound discussions among students a very reach data were collected (the results from qualitative parts of this study are under publication in another manuscript). Then the summary of discussions were presented in the meeting for getting the final agreement from the group members on the content of the meeting and the data collected. In the next step, a structured questionnaire was developed using the list of student challenges obtained from the previous stage, with the aim of quantifying every possible challenge. Face and content validity of the questionnaire was checked by experts from various health-related backgrounds. Then, a test-retest method was carried out to examine the reliability of the questionnaire with 10 students administering the test to the same group of individuals on two occasions in one week interval, reaching to 0.83 of reliability coefficient. A pilot study was carried out with 20 students, before main data collection, to make sure for any changes required either in the questions or in the process of data collection.

The questionnaire comprised two parts; (1) the students’ sociodemographic characteristics including: gender, age, marital status, having children, country of origin, major, educational level, school at TUMS, length of spent stay at TUMS, earlier university degree, and place of residency (e.g. dormitory or outside dorm); and (2) the items related to challenges facing the international students. Totally, the questionnaire consists of 40 items; 11 were related to participants’ characteristics, 29 items on a 5-point Likert scale (1 = never, 2 = rarely, 3 = neutral, 4 = sometimes, and 5 = frequently). Drawing on the standardized items, Cronbach’s alpha was applied to measure internal consistency was about 0.76.

The data were collected by two interviewers, both from recently graduated international students, with fluent English using probability proportional to size (PPS) sampling method, from all the schools and international student dormitories based on the number of all eligible students in each. Of the 218, 165 students have completed the questionnaire on a self-administered basis, thereby, the response rate was about 76%.

### Data analysis

The collected data were analyzed following assessment of data normality by the two-sample Kolmogorov-Smirnov test. Descriptive statistics (frequency, percentage, mean, and standard deviation), relationship between the challenges experienced by international students and their individual characteristics were identified using parametric tests including Pearson Correlation test, independent T-test and ANOVA, using SPSS 22.0 (SPSS Inc., Chicago, IL, USA). For all tests, results were considered statistically significant at *p* value ≤ 0.05.

## Results

### Students’ characteristics

As shown in Tables 1, 165 international students have completed the questionnaire. The median age for participants was 24.0 years (SD = 4.6); 49.1% were female; the vast majority were single (86.4%), therefore, 90.9% were with no children. The students were from among 16 different countries, however, 50.9% came from India and 11.5% were from Nigeria; 70.9% were studying medicine; 80.6% were undergraduates while about 20% were enrolled in graduate courses. Most of the participants (72.1%) have spent more than 3 years in Iran; 76.4% with no earlier university degree; 89.1% were living in the dormitory whilst 9.1% were renting.

**Table 1:** Description of sociodemographic characteristics of study participants* (*n* = 165)

Variable			Number– Percentage
**Age (years)**	Median = 24.00 SD = 4.587 years		
**Gender**	Male		84 (50.9)
	Female		81 (49.1)
**Marital status**	Single		143 (86.7)
	Married and living with spouse		14 (8.5)
	Married away from spouse		6 (3.6)
	Others		2 (1.2)
**Having any child**	Yes		15 (9.1)
	No		150 (90.9)
**Country of origin**	India		84 (50.9)
	Nigeria		19 (11.5)
	Ghana		6 (3.6)
	Others		56 (34)
**Major**	Medical Doctor		117 (70.9)
	Dentistry		13 (7.9)
	Others		35 (21.2)
**Educational level**	Bachelor		133 (80.6)
	Master		14 (8.5)
	Doctor of Philosophy		17 (10.3)
	Short course		1 (0.6)
**School of study at TUMS**	Medicine		119 (72.1)
	Dentistry		13 (7.9)
	Public Health		10 (6.1)
	Nursing		7 (4.2)
	Others		16 (9.7)
**Length of study at TUMS**	< 1 year		11 (6.7)
	2–3 years		35 (21.2)
	> 3 years		119 (72.1)
**Having earlier university degree**	Yes		39 (23.6)
	No		126 (76.4)
**Place of residence**	Dormitory		147 (89.1)
	Renting a house		15 (9.1)
	Owing a house		2 (1.2)
	Others		1 (0.6)

*Descriptive statistics

### Challenges facing international students at TUMS

As shown in Table 2, findings revealed that students face a number of challenges which were classified into three categories; academic, administrative, and socio-cultural challenges. By focusing on separate items, we found that poor updating of information provided by the university website (35.8% frequently, 21.2% sometimes), misleading information provided by staff (29.7% frequently, 26.1% sometimes), lack of guidance and orientation for new students (37% frequently), ineffective way of teaching Persian language (32.7% frequently, 19.4% sometimes), delay in replying to emails and poor online communication (35.2% frequently), low level of awareness and performance of staff (39.4% frequently), inability of staff to speak fluent English (33.3% frequently), poor accountability of university officials (44.2% frequently), time-consuming processes related to admission and graduation (41.8% frequently), lack of written guidelines and policies (32.7% frequently, 25.5% sometimes), changing policies in the midway of educational programs (56.4% frequently), and immature administrative system for international student affairs (37% frequently).

**Table 2:** Frequency and percentage of challenges experienced by international students* (*n* = 165)

Below is a list of possible challenges which have been experienced by students at TUMS. Please HONESTLY try to choose the most relevant answer for each item.	Never	Rarely	Neutral	Sometimes	Frequently
	N (%)				
I. **Academic challenges**					
1. Inability of university staff to speak fluent English	4(2.4)	29(17.6)	26(15.8)	51(30.9)	55(33.3)
2. Changing some polices and rules in the midway of the educational program	5(3.0)	9(5.5)	29(17.6)	29(17.6)	93(56.4)
3. Educational programs do not start and finish in the due time	23(13.9)	29(17.6)	38(23.0)	32(19.4)	43(26.1)
4. Difficulties in publishing articles in the European and American journals	21(12.7)	11(6.7)	72(43.6)	31(18.8)	30(18.2)
5. Lack of English language fluency of some instructors and lecturers	7(4.2)	26(15.8)	35(21.2)	45(27.3)	52(31.5)
6. Poor quality of educational content as well as lack of related assistance programs	28(17.0)	37(22.4)	45(27.3)	25(15.2)	29(17.6)
7. Low level of knowledge, skills and behavior of some professors	50(30.3)	44(26.7)	42(25.5)	18(10.9)	11(6.7)
8. Weak of scientific and educational content in program courses	44(26.7)	39(23.6)	46(27.9)	20(12.1)	16(9.7)
9. The weak international relations between TUMS and other universities (e.g. exchange programs)	15(9.1)	17(10.3)	51(30.9)	32(19.4)	50(30.3)
10. The continuous reduction of scholarships and limited financial support	8(4.8)	13(7.9)	45(27.3)	27(16.4)	72(43.6)
II. **Administrative challenges**					
1. The poor updating of information given by the university website	13(7.9)	15(9.1)	43(26.1)	59(35.8)	35(21.2)
2. The misleading information provided by the staff which might result in improper decisions made by the student	15(9.1)	19(11.5)	39(23.6)	49(29.7)	43(26.1)
3. Lack of guidance and orientation of the new students at arrival to the country	15(9.1)	17(10.3)	41(24.8)	31(18.8)	61(37.0)
4. The delay in replying to emails and poor online communication	12(7.3)	20(12.1)	38(23.0)	37(22.4)	58(35.2)
5. The low level of awareness and performance of the staff on matters related to the international students	8(4.8)	15(9.1)	32(19.4)	45(27.3)	65(39.4)
6. Poor accountability of university officials regarding international students’ issues	8(4.8)	13(7.9)	32(19.4)	39(23.6)	73(44.2)
7. The time-consuming processes related to the admission and graduation of international students	14(8.5)	14(8.5)	29(17.6)	39(23.6)	69(41.8)
8. Lack of written guidelines, policies, rules and regulations related to the affairs of international students	18(10.9)	19(11.5)	32(19.4)	42(25.5)	54(32.7)
9. Lacking announcement about the new rules and regulations	10(6.1)	21(12.7)	29(17.6)	34(20.6)	71(43.0)
10. Immature administrative system for international student affairs	13(7.9)	16(9.7)	36(21.8)	39(23.6)	61(37.0)
11. Problems in the proposal submission process and dissertation work, and the inconvenient communication with systems like Pajoheshyar and Sipad systems	12(7.3)	21(12.7)	48(29.1)	44(26.7)	40(24.2)
12. Poor access to internet in the university dormitories of international students	19(11.5)	29(17.6)	47(28.5)	39(23.6)	31(18.8)
III. **Socio-cultural challenges**					
1. Improper communication and coordination among employees in the offices related to the affairs of international students	7(4.2)	20(12.1)	29(17.6)	50(30.3)	59(35.8)
2. The ineffective way of teaching Persian language especially as an educational program language	31(18.8)	18(10.9)	54(32.7)	30(18.2)	32(19.4)
3. Discrimination in communication with international students	23(13.9)	22(13.3)	41(24.8)	37(22.4)	42(25.5)
4. Absence of extracurricular activities which is necessary for mental health and stress release	15(9.1)	18(10.9)	37(22.4)	47(28.5)	48(29.1)
5. Lack of counseling and support system for international students especially on some specific programs such as COVID-19 epidemic updates	32(19.4)	22(13.3)	46(27.9)	36(21.8)	29(17.6)
6. Lack of awareness on cultural diversity which may result in different types of conflict among students	22(13.3)	21(12.7)	60(36.4)	29(17.6)	33(20.0)
7. Lack of orientation sessions on the life outside the campus	13(7.9)	13(7.9)	43(26.1)	38(23.0)	57(35.2)

TOTAL (Range 0–116) *N* = 165, M = 71.84, SD = 21.93, Med = 73, Min = 5, Max = 116

* Descriptive statistics

Moreover, the students reported difficulties in proposal submission process and dissertation work, and inconvenient communication with systems like Pajoheshyar and Sipad systems (systems for checking academic performance and the progress in some educational-related processes) (26.7% frequently, 29.1% sometimes), difficulties in publishing articles in European and American journals (43.6% frequently), continuous reduction of scholarships and limited financial support (43.6% frequently, 27.3% sometimes), lack of English language fluency of some instructors and lecturers (31.5% frequently, 27.3% sometimes), poor quality of educational content as well as lack skills, and behavior of some professors (30.3% frequently, 26.7% sometimes), weak scientific and educational content in program courses (27.9% frequently, 23.6% sometimes), and poor access to the internet in university dormitories for international students (29.4% frequently, 25.4% sometimes).

Overall, our study showed that international students at TUMS face many challenges related to administrative, academic, and communication systems of the university. These findings suggest the need for the university to improve its services for international students and implement effective policies to address the identified challenges.

### Relationship between students’ characteristics and the total challenges

Table 3 explores the relationship between the total challenges experienced by the student and individual characteristics of the participants. The individual characteristics examined include age, gender, marital status, having children, major, degree, length of study in TUMS, earlier university degree, and place of residence.

**Table 3:** Relationship between students’ characteristics and total challenges they experienced*

Variable	N	Mean (SD)	*P*-Value (Significance)
Age	165	0.10	0.18
**Gender**			
Male	84	72.53 (22.49)	0.68
Female	81	71.12 (21.44)	
**Marital Status**			
Single	143	71.60 (21.98)	0.77
Married and living with spouse	14	71.21 (21.90)	
Married but living far from spouse	8	77.25 (23.24)	
**Having any child**			
Yes	15	69.73 (23.39)	0.69
No	150	72.05 (21.85)	
**Major**			
Medical doctor	117	73.76 (21.87)	0.10
Dentistry	13	69.92 (18.67)	
Others	35	66.14(22.74)	
**Educational level**			
BSc/MD/DDS	134	73.14 (21.80)	0.17
MSc	14	61.85 (18.58)	
PhD	17	69.76 (24.34)	
**Length of study in TUMS**			
1 year	11	47.00 (15.47)	< 0.001*
2–3 years	35	68.71 (23.22)	
> 3 years	119	75.05 (20.54)	
**Having earlier university degree**			
Yes	39	68.84 (21.29)	0.31
No	126	72.92 (22.10)	
**Place of residence**			
Dormitory	147	72.56 (22.31)	0.61
Renting a house	15	67.06 (19.44)	
Owing a house	2	57.00 (5.5)	

*relationship between age and report of challenges was examined by Pearson Correlation test, and others, based on being in two or three categories was examined by independent T-test and ANOVA

The results showed that among all the characteristics of students, only length of study in TUMS was significantly related to total challenges experienced, with participants studying for about a year having the lowest mean score, while those studying for more than three years had the highest mean score (*P* **< 0.001)**. Other characteristics did not show any significant relationships with the amount of challenges to be experienced.

Overall, these findings provide insights into the individual characteristics that may be associated with the challenges experienced by students in a medical university. However, the study has some limitations, including a small sample size for some subgroups and a lack of information on other potential factors that may influence challenges experienced, such as socioeconomic status, academic performance, and social support. Future studies could address these limitations and further explore the complex factors that contribute to the challenges experienced by medical students.

## Discussion

The present study aimed to identify the challenges facing international students at TUMS. The findings of the study revealed that international students at TUMS face various challenges, including academic, financial, social, cultural, and administrative challenges.

The academic challenges faced by international students at TUMS include difficulties in understanding the Persian language, accessing academic resources and support services, and coping with the rigorous academic curriculum. Many international students reported that they struggled to find textbooks and materials in their native language, which made it challenging for them to comprehend the course content. Regarding language barriers, previous studies highlighted that international students often struggle with language proficiency, which affects their academic performance and engagement [10]. Moreover, the lack of academic counseling or tutoring services for international students added to their academic challenges [11].

Financial constraints were also a significant challenge for international students at TUMS. Tuition fees for international students are generally higher than those for domestic students, and there are rather limited scholarship opportunities available to them. Furthermore, the high cost of living in Iran, especially in major cities like Tehran, added to the financial burden of international students, making it challenging for them to afford basic necessities such as housing and food. This also might negatively affect their wellbeing as stated in some studies [12]. In his study, Jonbekova pointed out that scholarship options are often limited for international students, hindering their ability to secure financial support for their studies [13].

However, the findings of this study also emphasize some special challenges faced by international students at TUMS, such as difficulty accessing academic resources and support services. As suggested in a qualitative study undertaken in public universities in Tehran, this may be due to the limited institutional support available to international students in Iranian universities. This underscores the need for universities in Iran to provide better support and resources to international students, in order to ensure their academic success and well-being [14].

Administrative challenges were also reported by international students at TUMS. Navigating the visa issues process was found to be challenging for many international students, with some experiencing delays or denials in their visa applications. Additionally, Iranian universities may not have the same level of institutional support for international students as universities in other countries, which can make it challenging for students to navigate the administrative and bureaucratic processes involved in studying abroad. Similar challenges were shown in an earlier study conducted in Portugal [15].

The social and cultural adjustment was another challenge reported by international students at TUMS. Iranian culture may be vastly different from the culture of their home country, and this can make it challenging for international students to integrate into the local community and make friends. The strict dress codes, social etiquette, and religious practices may vary from what international students are accustomed to, leading to feelings of isolation or confusion. In a study about the experience of Iranian students in Finland universities, Hosseini found that Finnish culture, and communication issue associated with language barriers and lack of job opportunities increase participants’ anxiety/uncertainty [16]. Similarly, an action research found that international students studying in the UK faced challenges related to language barriers, cultural differences, and academic support [17].

The findings of the present study suggest that the university ought to introduce more support to international students to help them overcome these challenges [15, 18, 19]. Persian language classes can be carried out to international students, however, academic counseling and tutoring services need to be provided in languages other than Persian, in addition, more scholarship opportunities need to be offered to alleviate financial constraints. TUMS can also establish cultural exchange programs to promote cross-cultural understanding and integration. In this regard, one of the several measures is to establish academic exchanges and joint training programs [20].

Despite these challenges, international students in Iranian universities also have opportunities for personal and academic growth. For example, they may have the chance to learn about a new culture, develop new skills, and build their academic and professional networks. Aydin (2020) suggested that Turkish universities can take steps to support international students, such as by providing language and cultural support programs and creating opportunities for social interaction [21].

This study witnessed a number of limitations; firstly, its cross-sectional nature limit for any further conclusion about the relationships between individual characteristics and the report of experienced challenges by them. Secondly, this study were during the Covid19 pandemic, thus the study phases and in particular data collection took too long and with special considerations for preventing virus transmission. However, as the first study conducted at TUMS for this topic and one of the first ones in whole country, the findings of this study can highly be practically applicable for conducting effective interventions by university managers for removing or at least limiting the reported experienced challenges by international students.

## Conclusion

International students at TUMS face various challenges related to academics, finance, social and cultural adjustment, and administrative issues. These challenges can negatively impact their academic performance, mental health, and overall experience of studying abroad. While the challenges faced by international students may vary depending on the country and university they are studying in, there are some common themes that emerge across different contexts. The findings of this study highlight the need for TUMS to provide more support to international students to help them overcome these challenges, such as language classes, academic counseling and tutoring services, and more scholarship opportunities. Moreover, TUMS can establish cultural exchange programs to promote cross-cultural understanding and integration. Iranian universities, in general, need to provide better support and resources to international students, in order to ensure their academic success and well-being and create a more welcoming and supportive environment for students from around the world. By addressing these challenges and leveraging the opportunities available to them, Iranian universities can attract more international students and contribute to their personal and academic growth while fostering a more diverse and inclusive learning community.

## Data Availability

The datasets used and/or analyzed during the current study available from the corresponding author on reasonable request.
